# Review on Symptomatic pedunculated leiomyomas in pregnancy with special consideration of an example case

**DOI:** 10.1007/s00404-024-07840-4

**Published:** 2024-11-27

**Authors:** Jonas Bubmann, Carl Mathis Wild, Christian Dannecker, Manuela Franitza, Bernadette Eser, Marina C. Seefried, Thomas Kroencke, Philipp Voisard, Udo Jeschke, Fabian Garrido

**Affiliations:** 1 Gynecology, Faculty of Medicine, University of Augsburg, Stenglinstrasse 2, 86156 Augsburg, Germany; 2 Bayerisches Zentrum für Krebsforschung (BZKF), Partnerstandort Augsburg, Augsburg, Germany; 3 Diagnostic and Interventional Radiology, Faculty of Medicine, University of Augsburg, Stenglinstrasse 2, 86156 Augsburg, Germany

**Keywords:** Myomectomy, Pregnancy, Pedunculated myoma, Fibroids, Laparotomy, Laparoscopy

## Abstract

**Objectives/Hypothesis:**

Symptomatic pedunculated leiomyomas in pregnancy; review of the literature with special consideration of an example case.

**Study design:**

Retrospective narrative review with an example case.

**Methods:**

Systematic evaluation of 37 reports.

**Example case:**

A 36-year-old Caucasian primigravida was referred symptomatic at 16 + 0 weeks due to a 13,5 cm myoma causing pain, constipation, urine retention and dysesthesias. Our patient underwent myomectomy at 17 + 0 weeks. One pedunculated leiomyoma was successfully removed.

**Conclusion:**

Myomectomy can be performed and is safe for pedunculated fibroids in pregnancy. Depending on the clinical scenario, surgical removal may be indicated. Based on the size of the fibroids and expected adhesions, a laparotomy is a safe option and is not a contraindication for vaginal birth in the case of pedunculated fibroids. Myomas larger than 10 cm should be removed by laparotomy.

## Introduction

Leiomyomas are the most common (20–40%) benign disease affecting the female reproductive system. The prevalence of leiomyomas in pregnancy is 2–10% and is usually asymptomatic, but 10% of patients develop complications in pregnancy [1]. Pain is often seen in women with fibroids > 5 cm [2]. In early pregnancy the volume of fibroids increases by 12% in volume because of the rapid increase in serum chorionic gonadotropin levels [3]. Only 37 pedunculated fibroids during pregnancy with single myomectomy are reported in the literature [4].Complication risk does increase with the number of fibroids, size, relation to the placenta and location [4]. Pain is related to the blood supply of the fibroid because increased growth can result in insufficient blood supply followed by necrosis [2].

### Example case presentation

**Fig. 1:**
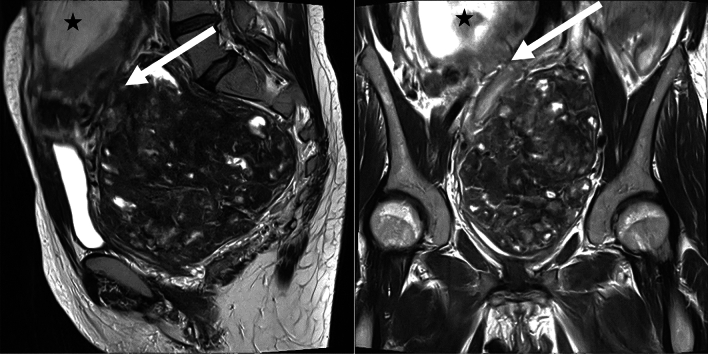
Preoperative MRI. T2-weighted images in the sagittal and coronal plane depicts a pedunculated sub serosal Leiomyoma (arrowhead) and the fetus (asterisk). The vascular pedicle visualized by worm-like signal-free vessels (arrowhead) suggests an attachment side of the leiomyoma to the posterior wall of the uterus. Degenerative changes (white spots) are seen within the leiomyoma

A 36-year-old primigravida presented to our University Hospital in January 2024 with a one-year history of chronic hematochezia, which had been treated with oral iron supplementation. The patient had been in the 16 + 0 weeks of gestation with a viable fetus (single, intra-uterine). The patient reported flank pain and micturition disorder. Diagnostic work-up included transabdominal ultrasound which identified a previously unknown pelvic mass. MR imaging was performed for better delineation and characterization of the mass. A 13.5 × 13 cm measuring pedunculated sub-serosal leiomyoma was diagnosed, originating from the posterior wall of the uterus. The lower abdominal organs had been shifted upwards with a compression-related urinary stasis III° on the left side and a compression of the common iliac vein (Fig. 1).

The uterine leiomyoma had not been diagnosed before the pregnancy, because the patient has not undergone regular screenings. The pregnancy had been developing physiological. After interdisciplinary discussion prophylactic low molecular weight heparin was prescribed. The patient developed dysesthesia in the left leg in the following weeks. Because of the increasing symptoms a conservative vs. surgical therapy was discussed with the patient. Double-J catheters had been inserted prior to surgery. Because of declining hemoglobin levels, two erythrocyte concentrates had been transfused.

**Fig. 2:**
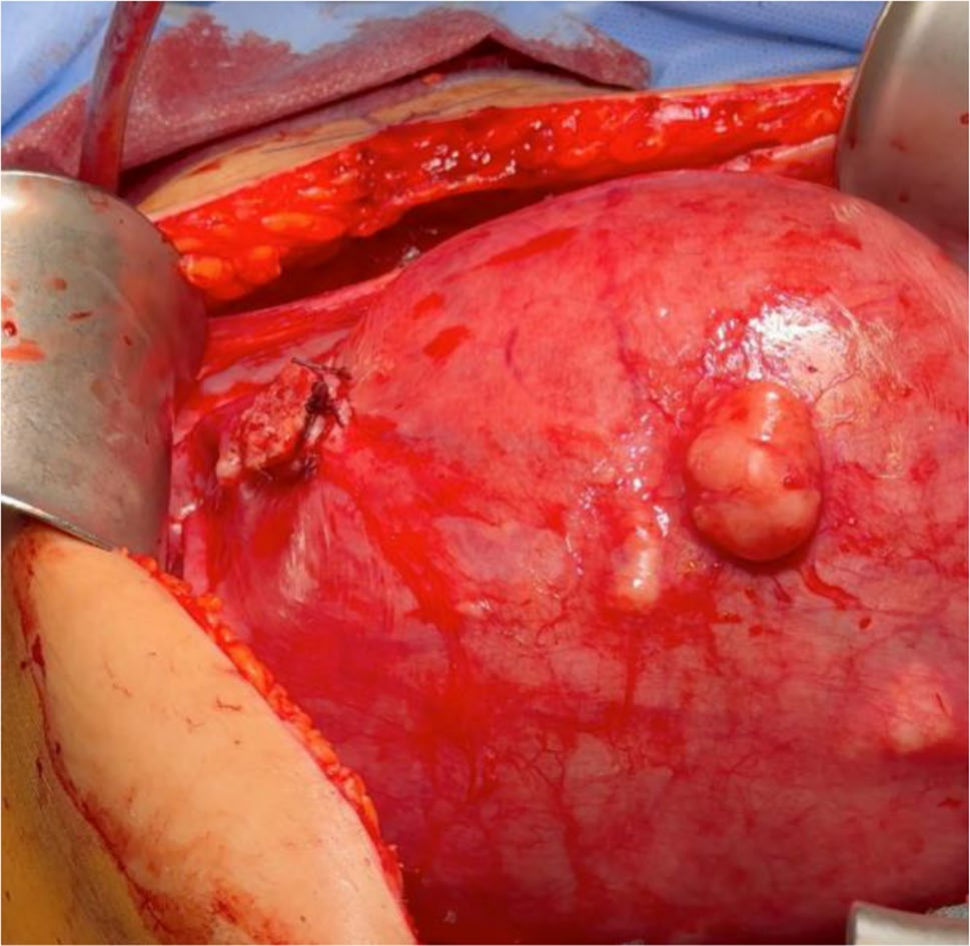
Stump of the pedicle

**Fig. 3:**
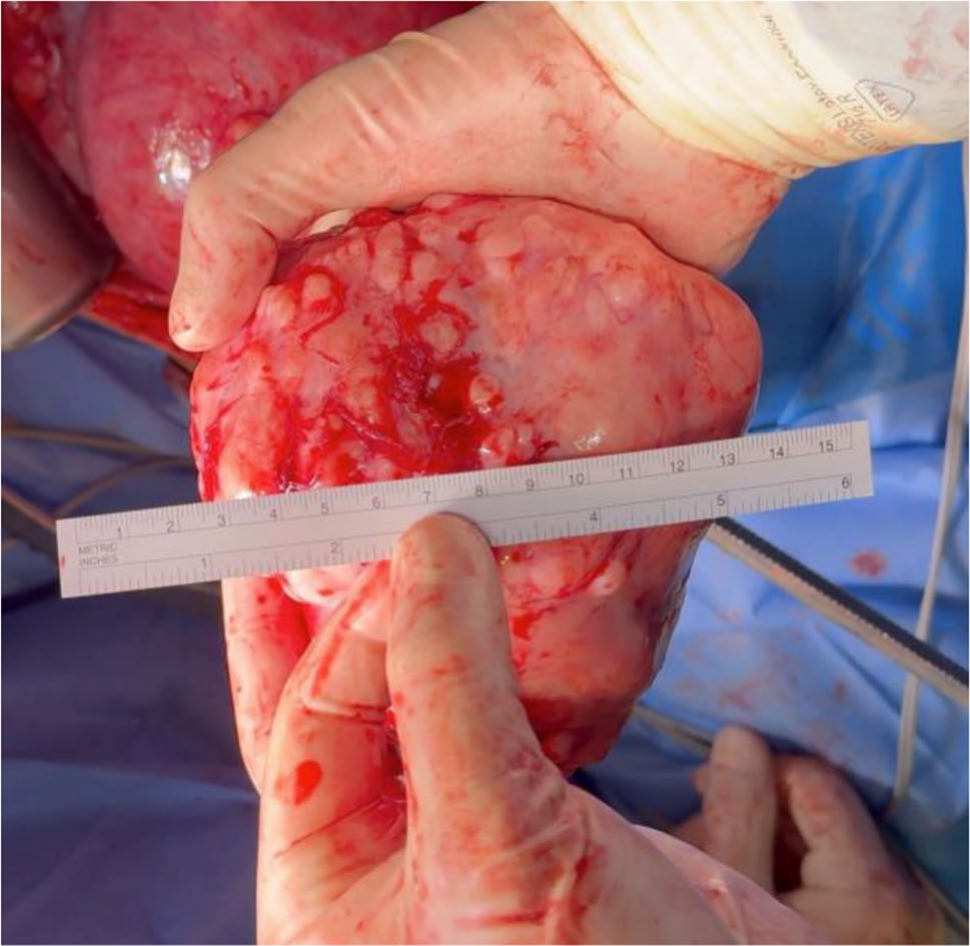
Myoma

On 24/01/2024 a longitudinal laparotomy was performed under general anesthesia with endotracheal intubation. Operative findings included normal liver, spleen, kidneys, diaphragm, ovaries and fallopian tubes. The uterus was soft and the size was adequate for 17 + 0 weeks of gestation. Fetal movements were visible. A pedunculated fibroid without a torsion measuring 13,5 cm in diameter filled out the whole lower sacral cavity. The pedicle was originating from the dorsal uterus with a stalk diameter of 2 cm. The fibroid was adherent to the peritoneum and sigmoid colon. It was detached and the pedicle was cut after ligation with several sutures 2-0 vicryl. After myomectomy, the pedicle was found to originate from the anterior wall of the uterus and had been turned during pregnancy dorsally. The estimated blood loss was 400 ml and the time of the surgery was 85 min. The tumor weighted 740 g and was sent for pathology. Pre-, intra- and postoperative performed sonographic vital controls of the fetus revealed normal findings (Fig. 2, 3).

Postoperatively, the patient was given indomethacin prophylactically to prevent uterine contractions. In the Further course, there was a slight neuropathy of the left leg, which after further diagnostics using MRI was most likely interpreted as the expression of an irritation caused by the pressure of the fibroid. There were no further abnormalities.

The symptoms improved and the patient was discharged from the hospital 10 days after the operation. The histological findings showed a leiomyoma with regressive changes and focal ischemic necrosis. There was no evidence of malignancy. The patient was followed up by us twice, and the pregnancy developed normally with no further complaints. Unfortunately, the patient decided to give birth in a rural hospital at 38 + 1 weeks’ gestation. Due to her history, a spontaneous labor was not performed and a secondary caesarean section was performed. A 3300 g baby with APGAR 9–10-10 was delivered. The patient was discharged after 4 days without complications.

The patient’s written consent to publication has been obtained.

## Review of literature

### Material and methods

**Fig. 4:**
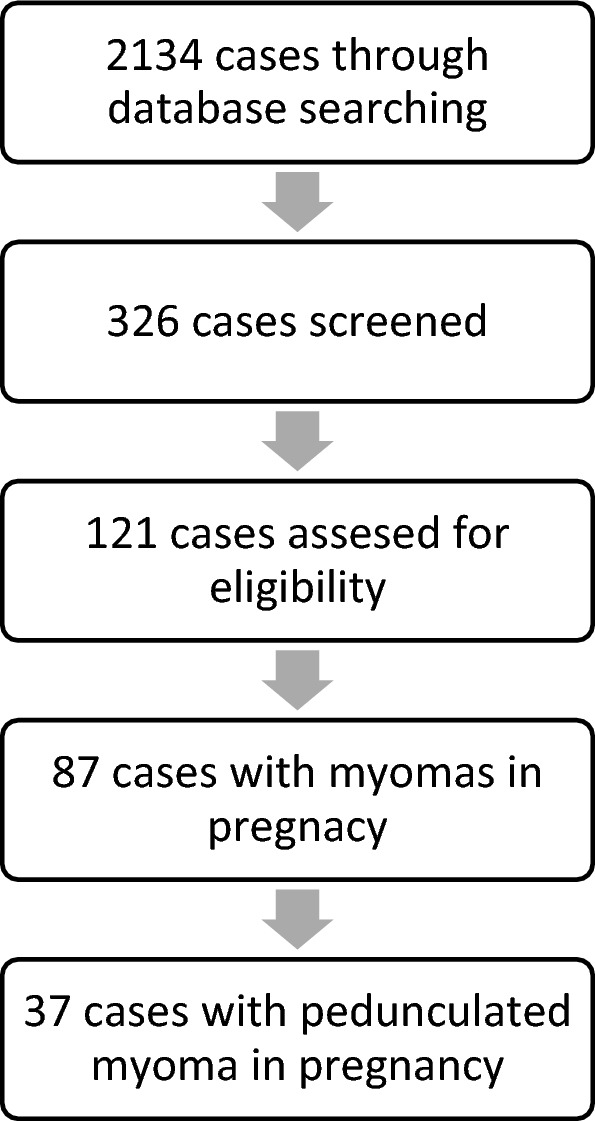
Overview of the review process

In March 2024, the search was carried out with various search terms in *Pubmed*^®^ (Table 1). The initial search was for case reports and case descriptions of fibroids during pregnancy. Cases with myomectomy during cesarean section were excluded. The cases were then reduced to pedunculated fibroids. Vaginally pedunculated fibroids were excluded. A table was created and the following parameters were recorded for each case: Year of treatment, name of author, title of publication, age of patient, parity, week of pregnancy at first presentation, week of pregnancy at treatment, location of fibroid, symptoms, sonographic findings, MRI findings, size of fibroid, type of operation, special features of operation, blood loss, antibiotics, tocolysis, pneumoperitoneum, intraoperative torsion, pathological findings, weight of fibroid, time of discharge, type of birth, week of pregnancy at birth and other complications. The data collection was carried out by one author (B.J.) (Fig. 4).

**Table 1:** Presentation of cases used for this review

Author	Date	Age	Pregnancy	Gestational week of surgery	Location of myoma	Symptoms	Imaging	Type of operation	Myoma weight	Size of myoma	Type of birth	Week of birth
Makar et al.	1989			14	Posterior wall	Pelvic pain	Sonography			120 mm	Vaginal	
Kalantaridou et al.	1994	38		19	Fundus	Pelvic pain	Sonography	Longitudinal laparotomy	1500 g			37
Pelosi et al.	1995	35	Primipara	13	Fundus	Pelvic pain	Sonography	LSK, free morcellation into abdominal cavity	1500 g	60 mm	Cesarean section for breech presentation	39
Sciannameo et al.	1996	31		20			MRI					
Majid et al.	1997	35	Multipara	17	Fundus	Gastrointestinal symptoms	Sonography			240 mm	Intrauterine fetal death	19
Luxman et al.	1997	27	Primipara	15	Fundus		Sonography	LSK, free morcellation into abdominal cavity		80 mm	Vaginal	39
Kalantaridou et al.	1999	25		16	Fundus	Pelvic pain	Sonography		170 g			39
Wittich et al.	2000	31	Primipara	15	Fundus	Pelvic pain	Sonography + MRI	Longitudinal laparotomy	2074 g	205 mm	Elective cesarean section	37
Kalantaridou et al.	2001	25		16	Anterior wall	Pelvic pain	Sonography + MRI		625 g			39
Sentilhes et al.	2003	35	Multipara	17	Left lateral wall	Pelvic pain	Sonography	LSK, free morcellation into abdominal cavity		50 mm	Elective cesarean section	37
Melgrati et al.	2005	29	Primipara	24	Fundus	Pelvic pain, fever	Sonography	Isobaric Laparoscopy		70 mm	Vaginal	39
Dracea et al.	2006	39	Multipara	14	Fundus		Sonography	Transverse Laparotomy		240 mm	Vaginal	37
Usifo et al.	2007	31	Primipara	13	Posterior wall	Pelvic pain, gastrointestinal symptoms	Sonography	Transverse Laparotomy		168 mm	Elective cesarean section	38
Okokwo et al.	2007	40		19	Fundus	Lower extremity edemas	Sonography	Longitudinal laparotomy	10,000 g	280 mm	Elective cesarean section	38
Leite et al.	2007	43	Primipara	17	Fundus	Pelvic pain	Sonography	Longitudinal laparotomy		91 mm	Vaginal	39
Alanis et al.	2008	22	Multipara	13	Fundus	Pelvic pain	MRI	Longitudinal laparotomy	8000 g	300 mm	Vaginal	38
Suwandinata et al.	2008	28	Primipara	15	Posterior wall	Pelvic pain	Sonography	Longitudinal laparotomy	320 g	80 mm	Elective cesarean section	37
Camacho et al.	2009	35	Multipara	16	Posterior wall	Pelvic pain, fever	Sonography	Longitudinal laparotomy		62 mm	Vaginal	40
Bhatla et al.	2009	30	Primipara	20	Fundus	Pelvic pain, gastrointestinal symptoms	Sonography	Longitudinal laparotomy	3900 g	280 mm	Vaginal	38
Fanfani et al.	2010	39	Primipara	25	Fundus	Pelvic pain	Sonography	LSK, Endo bag extraction	95 g	90 mm	Vaginal	40
Son et al.	2011	31	Primipara	18	Posterior wall		Sonography + MRI	LSK, Endo bag extraction	108 g	90 mm	Vaginal	39
Ardovino et al.	2011	31	Multipara	14	Fundus	Pelvic pain	Sonography	LSK, free morcellation into abdominal cavity	127 g	63 mm	Vaginal	40
Pelissier-Komorek et al.	2012	34	Primipara	10	Fundus	Pelvic pain, dyspnea	Sonography + MRI	Longitudinal laparotomy	2040 g	220 mm	Vaginal	35
Doerga-Bachasing et al.	2012	33	Multipara	10	Posterior wall	Pelvic pain, gastrointestinal symptoms	Sonography + MRI	Longitudinal laparotomy		175 mm	Cesarean section	40
Macció et al.	2012	33	Primipara	19	Fundus	Pelvic pain, gastrointestinal symptoms, vaginal bleeding	Sonography	LSK, Endo bag extraction	250 g	150 mm	Elective cesarean section with FGR	39
Macció et al.	2012	24	Primipara	20	Fundus	Pelvic pain	Sonography	LSK, Endo bag extraction	170 g	100 mm	Vaginal	40
Macció et al.	2012	34	Primipara	20	Anterior wall	Pelvic pain	Sonography	LSK, Endo bag extraction	240 g	40 mm	Vaginal	39
Tabandeh et al.	2012	30	Primipara	24	Fundus	Pelvic pain, gastrointestinal symptoms	Sonography + MRI	Longitudinal laparotomy		230 mm	Elective cesarean section	37
Currie et al.	2013	27	Primipara	11	Anterior wall	Pelvic pain	Sonography	LSK with Pfannenstiel incision		80 mm		
Domenici et al.	2013	35	Primipara	16	Posterior wall	Pelvic pain, urinary habit changes	Sonography + MRI	Longitudinal laparotomy		200 mm	Elective cesareans section	38
Saccardi et al.	2014	35	Primipara	15	Anterior wall	Pelvic pain, gastrointestinal symptoms	Sonography	LSK, free morcellation into abdominal cavity	1363 g	240 mm	Cesarean section for fetal tachycardia	41
Anthimides et al.	2015	31	Multipara	10	Fundus	Pelvic pain	Sonography	LSK, Endo bag extraction		77 mm		
Algara et al.	2015	36		18		Pelvic pain		LSK			Intrauterine fetal death after car accident	
Jhalta et al.	2016	34	Primipara	14	Fundus		Sonography	Longitudinal laparotomy		160 mm	Vaginal	39
Kim et al.	2016	35	Primipara	10	Fundus	Pelvic pain	Sonography	LSK		93 mm	Vaginal	41
Basso et al.	2017	36	Multipara	17	Anterior wall	Pelvic pain	Sonography	Longitudinal laparotomy		132 mm	Vaginal	38
Our case	2024	37	Primipara	18	Anterior wall	Pelvic pain, gastrointestinal symptoms, urinary habit changes	Sonography + MRI	Longitudinal laparotomy	740 g	135 mm	Secondary caesarean section	39

### Statistics

The statistical analysis was performed using data from eligible studies to assess the overall effect sizes and heterogeneity. Effect measures, such as odds ratios (OR) and risk ratios (RR), with 95% confidence intervals (CI), were calculated for binary outcomes, while mean differences (MD) were used for continuous outcomes. Funnel plots and Egger’s regression test were conducted to assess potential publication bias, with a *p*-value < 0.05 considered statistically significant.

## Results

2134 cases were found and 326 of them further screened. 121 cases had been eligible, but only 87 of them had myomectomies during pregnancy. All cases before the introduction of ultrasound diagnostics were excluded. The oldest included case was from 1989 [5], seven cases were before 2000 [6–10], twelve cases between 2000 and 2010 [6, 11–21] and 17 cases since 2010 to date [22–36]. The most recent case was from 2017 [36]. 37 cases reported about pedunculated myomas during pregnancy. One study was translated into Spanish. The remaining cases were in English. We include our above-mentioned case in the systematic review.

### Demography

The age was noted in 37 [6–23, 23–36] of the 38 cases. The average age was 32.7 years (± 4.72; 22–43 years). 55.3% (*n* = 21) were primipara [7, 10, 11, 13, 15, 17, 19, 21–23, 26, 28–31, 34, 35] and 26,3% (*n* = 10) of the patients had already been pregnant once [9, 12, 14, 18, 20, 25, 27, 32, 36], whereby no information was provided in 7 case reports [5, 6, 8, 16, 33].

### Symptomatology and diagnosis

On average, the patients presented at 14.97 weeks of pregnancy (± 4.38; range: 7–25). Only one patient [14] showed no symptoms at all and no information about the symptoms was reported in three patients [8, 10, 24]. 81.6% (*n* = 31) of the patients reported pelvic pain. In addition, 18.4% (*n* = 7) had gastrointestinal symptoms in combination or alone [9, 15, 21, 22, 27–29]. Urinary habit changes were reported in 5.3% (*n* = 2) of cases [31]. Fever, edema of the lower extremities or vaginal bleeding were also described in 2.6% (*n* = 1) [16, 20, 28].

71.1% (*n* = 27) of the patients had only a preoperative sonography [5–7, 9, 10, 12–17, 19–23, 25, 28, 30, 32, 34–36], with 5,3% (*n* = 2) having an MRI [8, 18] and only 18,4% (*n* = 7) having an MRI and sonography [11, 26–29, 31]. No examination was documented in one patient [33].

A pedunculated fibroid was diagnosed preoperatively in 14 ultrasound findings [5, 6, 14, 22, 25, 27, 28, 35], with 22 findings not describing a pedicle. 55.3% (*n* = 21) of the fibroids originated from the fundus, [6, 7, 9–11, 13, 14, 16–18, 21, 23, 25, 26, 28, 29, 32, 34, 35] followed by 21.1% (*n* = 8) from the posterior wall [5, 15, 19, 20, 27, 28, 31]. 15.8% (*n* = 6) were on the anterior wall [22, 28, 30, 36] and only one fibroid (2.6%) was lateral to the uterus [12].

A sub-serosal fibroid was described in all MRI examinations, [8, 11, 17, 24, 27, 29, 31] whereby a torsion could already be detected in one finding [8]. At least one further fibroid was diagnosed in 28,9% (*n* = 11) of the patients [6, 9, 14, 19, 26, 28, 31, 36]. 21% (*n* = 8) of patients showed multiple uterine fibroids [6, 9, 26, 28, 36].

### Management

No case report could be found that describes a wait-and-see approach.

47.4% (*n* = 18) of the patients underwent open surgery, [6, 11, 14–21, 26, 27, 29, 31, 34, 36] with a longitudinal laparotomy being performed in 42.1% (*n* = 16) of cases [6, 11, 16–21, 26, 27, 29, 31, 34, 36] and a transverse laparotomy in 5.3% (*n* = 2) [14, 15]. Laparoscopy (LSK) was performed in 39.5% (*n* = 15) [7, 10, 12, 13, 22–25, 28, 30, 32, 33, 35].

Significantly (*p* =  < 0.001), larger fibroids underwent laparotomy and smaller fibroids underwent LSK. There was also a significance (*p* = *0.018*) between the weight of the fibroid and the type of surgery. Patients who underwent open surgery had fibroids that were on average 181.71 mm (± 71.64 mm; 62–300 mm) in size and 3571.8 g (± 3556 g; 320–10.000 g) in weight. In contrast, the patients with LSK had 91.6 cm (± 50.13 cm; 40–240) measuring fibroids and lighter fibroids weighing 481.6 g (± 590.1 g; 95–1500 g). Surprisingly, the transverse laparotomy was more likely to yield heavy fibroids (204 g; ± 50.9 g) than longitudinal laparotomy (178.8 g; ± 78.4 g).

In only *8* patients the time between the first presentation and the surgical treatment documented [11, 18, 20–22, 27, 29]. For these, the average time span was 6.25 weeks (± 4.13; 1–12 weeks). We assume that the weeks of pregnancy stated in the case report also correspond to the time of surgical treatment. Thus, on average, the patients underwent surgical intervention at 16.3 weeks of pregnancy (± 3.78; 10–25). Blood loss was reported in *17* patients, [7, 9, 13, 15, 16, 19, 21, 22, 25, 27, 28, 28, 31, 35] with a mean value of 607 ml (± 1.076 ml; 0–4.500 ml) and thus a very wide range. Nevertheless, only one patient is described as requiring a postoperative transfusion [21]. No antibiotics were discussed in 17 patients, [5, 6, 8, 10, 13, 15–17, 26–29, 33] so that 26.3% (*n* = 10) received antibiotics [12, 14, 18, 19, 21, 31, 34, 36] and 28.9% (*n* = 11) did not receive antibiotics [8, 10, 12, 21, 23–25, 30, 32]. Only one patient was described as having a post-operative infection with abscess development at the uterine scar. [12] This one patient had a laparoscopy and did not receive antibiotics intraoperatively [12].

26.3% (*n* = *10*) patients received tocolysis pre- or postoperatively, [9, 11, 14, 17, 21, 29, 34, 36]. whereas 34.2% (*n* = 13) did not receive tocolysis [7, 12, 18–20, 22–25, 30–32, 35]. In 15 patients no information regarding tocolysis was documented [5, 6, 8, 10, 13, 15, 16, 26–28, 33]. No patient developed labor until postoperative discharge. Torsion of the fibroid was present in 26.3% (*n* = *10*) of the cases. [9, 11, 15, 21, 30, 32, 33, 35], However, the majority of 55.3% (*n* = *21*) had no torsion [6, 7, 11–13, 17, 19, 21–23, 25, 27–29, 31, 34]. Of the LSK patients, 4 underwent free morcellation into the abdominal cavity [7, 10, 12, 22, 25]. In 6 of the LSK patients, the fibroid was retrieved using an Endo bag [23, 24, 28, 32]. In one patient, attention was paid to isobaric pressure [13]. The intra-abdominal pressure during LSK was described in 11 patients [7, 10, 13, 19, 22–25, 28, 32] and was found to be 11.2 mmHg (± 1.687 mmHg; 10–14 mmHg) on average.

### Histopathology

In 31 operations, a histopathological examination was subsequently performed [5, 7, 9, 11–13, 15–26, 28, 30–36]. This revealed a degenerative change in the fibroid in 41.9% (*n* = 13) of the patients [6, 12–14, 16–20, 26, 28, 35]. 20 fibroids were described as sub serosal [9, 13, 15, 17, 19, 21, 23, 25–28, 30–36], whereby the other cases did not document any such description regarding the localization.

### Postoperative time

On average, patients were discharged 4.64 (± 2.99; 1–14) days after surgery.

81.3% (*n* = 31) had a complication-free postoperative course. Postoperative complications were described in only 10.5% (*n* = 4) of the patients [12, 21, 27, 36]. One patient developed an abdominal abscess [12], as mentioned above and one patient required a transfusion of two red blood cell coagulates [21]. One patient developed cervical insufficiency in the 21st week of pregnancy [36].

One child died in the immediate post-operative period after multiple myomas had been removed and an appendectomy was performed [9].

### Delivery

On average, all other cases had a birth in the 38.6 (± 1.36; 35–41) week of pregnancy. Of these, 47.2% (*n* = 17) had a vaginal birth [5, 10, 13, 14, 17, 18, 20, 21, 23–26, 28, 34–36]. A cesarean section was performed in 30.6% (*n* = 11) of cases [7, 11, 12, 15, 16, 19, 22, 27–29, 31]. No case reported a reduced APGAR or postpartum abnormalities. Significantly (*p* = 0.002), the patients with laparotomy gave birth at 37.9 (± 1.26; 35–40) weeks gestation, whereas the patients with LSK gave birth at 39.4 (± 1.08; 37–41) weeks gestation. There was no significance in regard to the type of birth.

## Discussion

Surgical interventions are avoided during pregnancy, if possible, but our case and review of the literature show that surgical interventions may be necessary in pregnant patients. Symptomatic pedunculated fibroids rarely occur in pregnancy but require a well prepared and considered treatment. Patients with the combination of sonography and MRI showed the lowest complication rate. This is also confirmed by the fact that visualization of a pedicle enabling a diagnosis of a pedunculated leiomyoma was mostly successful on MRI.

Our decision to perform open surgery was in line with the current literature. We conclude that fibroids larger than 10 cm should be treated by laparotomy. In addition, adhesions with the fibroid should always be considered and expected.

The complexity of our case, as well as the literature review, show that surgically experienced personnel are essential for these procedures. Even if there is a temptation to enucleate further fibroids, the cases to date confirm that only the symptomatic fibroid should be removed. Experienced anesthetists should be present, as blood loss of up to 4.5 L has been described. Patients receiving antibiotic therapy showed no further infection, which was also confirmed in our case. Transfusion is not often needed prior surgery [21]. Ligation of the stalk by means of vicryl sutures is the most commonly applied technique, followed by staplers and bipolar electrosurgical devices. Vaginal delivery mode is seen in single myomectomies by 30–45%, even though there is missing data about the pedunculated situation [37].

It is astonishing that neither in patients with tocolysis nor without tocolysis a labor induction kit was described in any case.

Torsion of a fibroid is rare but should be considered first, as further complications such as necrosis or septicemia can develop.

A primary cesarean section was not recommended in any of the cases. No complications were described in any of the vaginal births. This confirms previous literature that vaginal birth is possible after myomectomy without opening the uterine cavity [38].

## Conclusion

Myomectomy can be performed safely for pedunculated fibroids in pregnancy. MRI is helpful for fibroid mapping and maybe considered when sonography is insufficient. Based on the size of the fibroids and expected adhesions, a laparotomy is a safe option and is not a contraindication for vaginal birth in case of pedunculated fibroids. Myomas larger than 10 cm should be removed by laparotomy.
